# Development and external validation of an interpretable machine learning model for diagnosing coronary heart disease in patients with type 2 diabetes and MASLD

**DOI:** 10.3389/fendo.2026.1830594

**Published:** 2026-05-15

**Authors:** Chunxia Deng, Ling Feng, Tingting Li, Suosu Wei, Huiming Zhu, Jie Lu

**Affiliations:** 1 Department of Endocrinology and Metabolism, Guangxi Academy of Medical Sciences and the People’s Hospital of Guangxi Zhuang Autonomous Region, Nanning, Guangxi, China; 2 Department of Respiratory and Critical Care Medicine, Guangxi Hospital of the First Affiliated Hospital, Sun Yat-sen University, Nanning, Guangxi, China; 3 Clinical Research Center of Guangxi Academy of Medical Sciences, The People’s Hospital of Guangxi Zhuang Autonomous Region, Nanning, Guangxi, China; 4 Department of Clinical Laboratory, Nantong Sixth People’s Hospital Affiliated to Shanghai University, Nantong, Jiangsu, China

**Keywords:** coronary heart disease, external validation, machine learning, metabolic dysfunction-associated steatotic liver disease, SHAP, type 2 diabetes mellitus, XGBoost

## Abstract

**Introduction:**

Patients with type 2 diabetes mellitus (T2DM) and metabolic dysfunction-associated steatotic liver disease (MASLD) face substantially elevated coronary heart disease (CHD) risk, yet no machine learning diagnostic models exist specifically for this population. This study aimed to develop and validate an interpretable machine learning model for identifying CHD in T2DM-MASLD patients.

**Methods:**

Using data from 1,269 patients (development cohort) and 1,058 patients (external validation cohort) from two Chinese hospitals, we compared seven machine learning algorithms. Angiographically confirmed CHD served as the diagnostic endpoint. Nine features were selected by univariate analysis, LASSO regression, and the Boruta algorithm. The best-performing model was selected based on comprehensive evaluation of discrimination, calibration, and clinical utility. Model interpretability was assessed using SHapley Additive exPlanations (SHAP), and external validation was performed in an independent cohort.

**Results:**

Feature selection identified nine predictors: total cholesterol (TC), chest distress, apolipoprotein B (ApoB), male sex, triglycerides (TG), age, chest pain, red cell distribution width (RDW), and cardiac troponin (cTn). The XGBoost model achieved the best performance, with an AUC of 0.896 (95% CI, 0.862–0.930) in internal validation and 0.865 (95% CI, 0.837–0.893) in external validation, with excellent calibration (Brier score: 0.112). To facilitate clinical application, a freely accessible web-based calculator was developed for real-time individualized CHD risk prediction.

**Discussion:**

This is the first interpretable machine learning model externally validated for CHD diagnosis in T2DM-MASLD patients, demonstrating robust performance using nine routinely available clinical parameters. The model's interpretability through SHAP analysis enhances clinical trust and supports individualized risk communication between physicians and patients to guide decisions regarding coronary angiography.

## 1 Introduction

Type 2 diabetes mellitus (T2DM) represents a major global health challenge, with prevalence continuing to rise worldwide. Approximately 529 million adults had diabetes in 2021, and this number is projected to reach 1.31 billion by 2050 (1). Coronary heart disease (CHD) is the leading cause of death in patients with T2DM, accounting for approximately 50% of diabetes-related mortality (2). The coexistence of metabolic dysfunction-associated steatotic liver disease (MASLD, formerly known as non-alcoholic fatty liver disease, NAFLD) further exacerbates cardiovascular risk. Recent meta-analyzes indicate that the global prevalence of MASLD among T2DM patients is approximately 65–70%, with even higher rates observed in Asian populations (3, 4). In China, the prevalence of MASLD among T2DM patients is approximately 63.2%, representing a substantial high-risk population (5).

Notably, MASLD is now recognized not merely as a hepatic condition but as a multisystem metabolic disorder with profound cardiovascular implications. The 2025 American Diabetes Association consensus report emphasizes that “MASLD significantly increases the risk of developing T2DM, cardiovascular disease, and extrahepatic malignancies” (6). A recent meta-analysis encompassing nearly 12 million patients demonstrated that MASLD is associated with a 54% increased risk of cardiovascular events (HR 1.54, 95% CI 1.32–1.81) and a 46% increased risk of cardiovascular mortality (HR 1.46, 95% CI 1.40–1.51), with MASLD showing stronger associations with cardiovascular events than NAFLD (7). Importantly, diabetes combined with MASLD substantially increases the risk of subclinical atherosclerosis, with a 5.8-fold higher odds of coronary artery calcification compared to individuals without fatty liver disease (8).

Despite these well-established associations, accurately identifying patients with existing CHD among those with concurrent T2DM and MASLD (T2DM-MASLD) remains challenging, particularly in determining which patients warrant invasive coronary angiography. Traditional risk assessment tools, including the Framingham Risk Score (FRS), Pooled Cohort Equations (PCE), and the American Heart Association PREVENT equations, perform suboptimally in MASLD populations. A recent study from the TARGET-NASH cohort demonstrated that the FRS achieved a C-statistic of only 0.58 (95% CI 0.52-0.63) for predicting cardiovascular events in MASLD patients, while the PREVENT model achieved 0.60 (95% CI 0.54-0.65), with significant calibration drift across risk deciles (9). These findings highlight the urgent need for improved risk stratification tools specifically designed for this high-risk population.

Machine learning (ML) approaches offer promising solutions to overcome the limitations of traditional risk models. Unlike conventional statistical methods, ML algorithms can automatically identify complex nonlinear relationships and high-order feature interactions within clinical data, potentially uncovering novel risk patterns unique to T2DM-MASLD patients (10). Recent studies have demonstrated the superiority of ML models, particularly ensemble methods such as XGBoost and Random Forest, in diagnosing and predicting cardiovascular outcomes in diabetic patients (2, 11, 12). However, the “black-box” nature of these algorithms has limited their clinical acceptance, as physicians require transparent, interpretable predictions to guide clinical decision-making (13).

The emergence of explainable artificial intelligence techniques, particularly SHapley Additive exPlanations (SHAP), addresses this critical limitation. SHAP analysis provides both global feature importance rankings and individualized explanations of model predictions, enabling clinicians to understand how each clinical variable contributes to a patient’s risk assessment (13, 14). This transparency not only enhances clinical trust but also facilitates the identification of modifiable risk factors for targeted intervention. Studies have shown that SHAP-based interpretable models can achieve comparable or even superior predictive performance while maintaining clinical interpretability, making them suitable for integration into clinical decision support systems (15, 16).

To date, no study has specifically developed and validated an interpretable ML model for diagnosing CHD in Chinese patients with concurrent T2DM and MASLD. Given the unique pathophysiological interplay between hepatic steatosis, insulin resistance, and accelerated atherosclerosis in this population, dedicated diagnostic tools are urgently needed to guide decisions regarding invasive coronary angiography. This study aimed to: (1) develop and externally validate an interpretable machine learning model for diagnosing angiographically confirmed CHD in Chinese T2DM-MASLD patients using readily available clinical parameters; (2) employ SHAP analysis to identify and rank the most important diagnostic features; and (3) provide individualized risk explanations to guide clinical decision-making regarding further cardiovascular evaluation.

## 2 Methods

### 2.1 Study design and data source

This retrospective cohort study included patients with T2DM and concurrent MASLD hospitalized at the People’s Hospital of Guangxi Zhuang Autonomous Region between January 2012 and December 2020 for model development and internal validation. Patients from Nantong Sixth People’s Hospital were included as an external validation cohort. Inclusion criteria were: (1) confirmed diagnosis of T2DM according to the Chinese Diabetes Society guidelines or documented history of T2DM (17); (2) MASLD diagnosed according to the 2023 multi-society consensus criteria; (3) age ≥18 years; and (4) availability of coronary angiography results. Exclusion criteria were: (1) other types of diabetes or acute diabetic complications; (2) prior history of CHD or other cardiac diseases including congenital heart disease, rheumatic heart disease, or heart failure; (3) other liver diseases such as viral hepatitis, autoimmune hepatitis, or drug-induced liver injury; (4) significant alcohol consumption (≥140 g/week for women or ≥210 g/week for men); (5) malignant tumors; (6) autoimmune diseases such as systemic lupus erythematosus; (7) current use of hepatotoxic drugs; and (8) missing data exceeding 30% of total variables.

### 2.2 Data collection and definitions

Data from each patient’s first admission were extracted from the electronic medical record system, including:(1) demographic information: sex, age, smoking status, and history of hypertension; (2) clinical symptoms: polyuria, chest distress, chest pain, palpitation, hypodynamia, emesis, cold sweat, nausea, and fever; (3) laboratory tests: white blood cell count (WBC), neutrophil count, lymphocyte count, monocyte count, eosinophil count, basophil count, red blood cell count (RBC), hemoglobin (HGB), platelet count (PLT), red cell distribution width (RDW), triglycerides (TG), total cholesterol (TC), low-density lipoprotein cholesterol (LDL-C), high-density lipoprotein cholesterol (HDL-C), apolipoprotein A1 (ApoA1), apolipoprotein B (ApoB), total protein (TP), albumin (ALB), aspartate aminotransferase (AST), alanine aminotransferase (ALT), lactate dehydrogenase (LDH), gamma-glutamyl transferase (GGT), creatinine (Cr), urea, uric acid (UA), cardiac troponin (cTn), myoglobin (Myo), creatine kinase-MB (CK-MB), thyroid-stimulating hormone (TSH), free thyroxine (FT4), free triiodothyronine (FT3), activated partial thromboplastin time (APTT), prothrombin time (PT), thrombin time (TT), fibrinogen (Fbg), D-dimer (DD), C-reactive protein (CRP), glycated hemoglobin (HbA1c), and homocysteine (HCY).

MASLD was diagnosed according to the 2023 multi-society Delphi consensus nomenclature, defined as the presence of hepatic steatosis in patients with at least one of five cardiometabolic risk factors: (1) body mass index ≥25 kg/m² (≥23 kg/m² for Asians) or waist circumference >94 cm for men or >80 cm for women; (2) fasting serum glucose ≥5.6 mmol/L, HbA1c ≥5.7%, type 2 diabetes, or treatment for type 2 diabetes; (3) blood pressure ≥130/85 mmHg or antihypertensive drug treatment; (4) plasma triglycerides ≥1.70 mmol/L or lipid-lowering treatment; (5) plasma HDL-cholesterol ≤1.0 mmol/L for men and ≤1.3 mmol/L for women or lipid-lowering treatment (18).

### 2.3 Outcome definition

The primary endpoint was the presence of angiographically confirmed CHD at the time of index hospitalization. According to the American College of Cardiology/American Heart Association guidelines, CHD was diagnosed when coronary angiography revealed ≥50% stenosis in at least one major epicardial coronary artery (left main, left anterior descending, left circumflex, or right coronary artery) (19).

### 2.4 Sample size calculation

Sample size adequacy was assessed using the events per variable (EPV) principle. For binary outcome prediction models, a minimum of 10 events per candidate predictor variable is recommended to minimize overfitting and ensure stable coefficient estimates (20). With 238 CHD events and 9 final predictor variables in the training cohort, our study achieved an EPV of 26.4, exceeding the recommended threshold and supporting model stability.

### 2.5 Data preprocessing and feature selection

To ensure data completeness and accuracy, variables with greater than 30% missing values were excluded. For continuous variables, values exceeding 3 standard deviations were treated as missing. Missing values were handled using multiple imputation by chained equations (MICE) implemented via the “mice” package in R, with five random imputations performed according to variable type; numerical variables were imputed using the mean and categorical variables using the mode to generate the initial complete dataset. Continuous variables were standardized using z-score normalization prior to model training, with standardization parameters derived from the training set and applied to both validation sets to prevent data leakage.

The data were randomly split into training (70%) and internal validation (30%) sets using stratified sampling to maintain consistent CHD event proportions. In the training set, feature variables were selected through univariate analysis, least absolute shrinkage and selection operator (LASSO) regression, and the Boruta algorithm. The intersection of variables selected by all three methods, combined with prior literature and expert opinion, was used to determine the features most relevant to the outcome.

### 2.6 Machine learning model development and evaluation

Seven machine learning algorithms were used to construct prediction models in the training set based on the selected features: logistic regression (LR), decision tree (DT), random forest (RF), extreme gradient boosting (XGBoost), light gradient boosting machine (LightGBM), support vector machine (SVM), and artificial neural network (ANN). Hyperparameter optimization was performed using grid search combined with 5-fold cross-validation on the training set. The final models were trained on the entire training set using the optimized hyperparameters.

Model performance was evaluated on the internal validation set using the following metrics. Receiver operating characteristic (ROC) curves, calibration curves, and decision curve analysis (DCA) were plotted for each model. The area under the ROC curve (AUC) was calculated to determine discriminative ability; calibration curves and Brier scores were used to assess agreement between predicted and observed outcomes; and DCA was used to evaluate clinical benefit and applicability. Additionally, accuracy, sensitivity, specificity, F1 score, positive predictive value (PPV), and negative predictive value (NPV) were calculated to evaluate model performance. The best-performing model was selected based on comprehensive evaluation for external validation to assess diagnostic generalizability across different clinical settings.

### 2.7 Interpretability of machine learning prediction model

SHAP is a game-theoretic approach that quantifies the contribution of each feature to individual predictions by calculating Shapley values. Feature importance ranking plots and beeswarm plots were generated to provide global explanations, while force plots were generated to provide local explanations for individual samples, enhancing model interpretability and applicability.

### 2.8 External validation

The generalizability of the best-performing model was evaluated using an independent external validation cohort from Nantong Sixth People’s Hospital. Inclusion and exclusion criteria were identical to those described above. Model performance was assessed using the same metrics (AUC, calibration curves, Brier score, and DCA) without any model recalibration or updating, representing a rigorous test of model transportability.

### 2.9 Web-based clinical calculator deployment

To facilitate clinical translation, the optimized XGBoost model was deployed as a freely accessible web-based clinical calculator using the Streamlit framework (Python). The application accepts the nine selected clinical parameters as input and automatically calculates the individualized CHD probability along with real-time SHAP-based visualizations (force plot and waterfall plot) that visually explain the contribution of each feature to the prediction.

### 2.10 Statistical analyzes

Statistical analyzes were performed using R version 4.5.1 and Python version 3.10.4. Continuous variables were tested for normality using the Shapiro-Wilk test. Normally distributed continuous variables were expressed as mean ± standard deviation, with between-group comparisons performed using t-tests; non-normally distributed continuous variables were expressed as median (interquartile range), with between-group comparisons performed using the Mann-Whitney U test. Categorical variables were expressed as frequencies (percentages), with between-group comparisons performed using chi-square tests or Fisher’s exact test when expected frequencies were less than 5. All tests were two-sided, and P<0.05 was considered statistically significant.

## 3 Results

### 3.1 Patient characteristics

**Figure 1:**
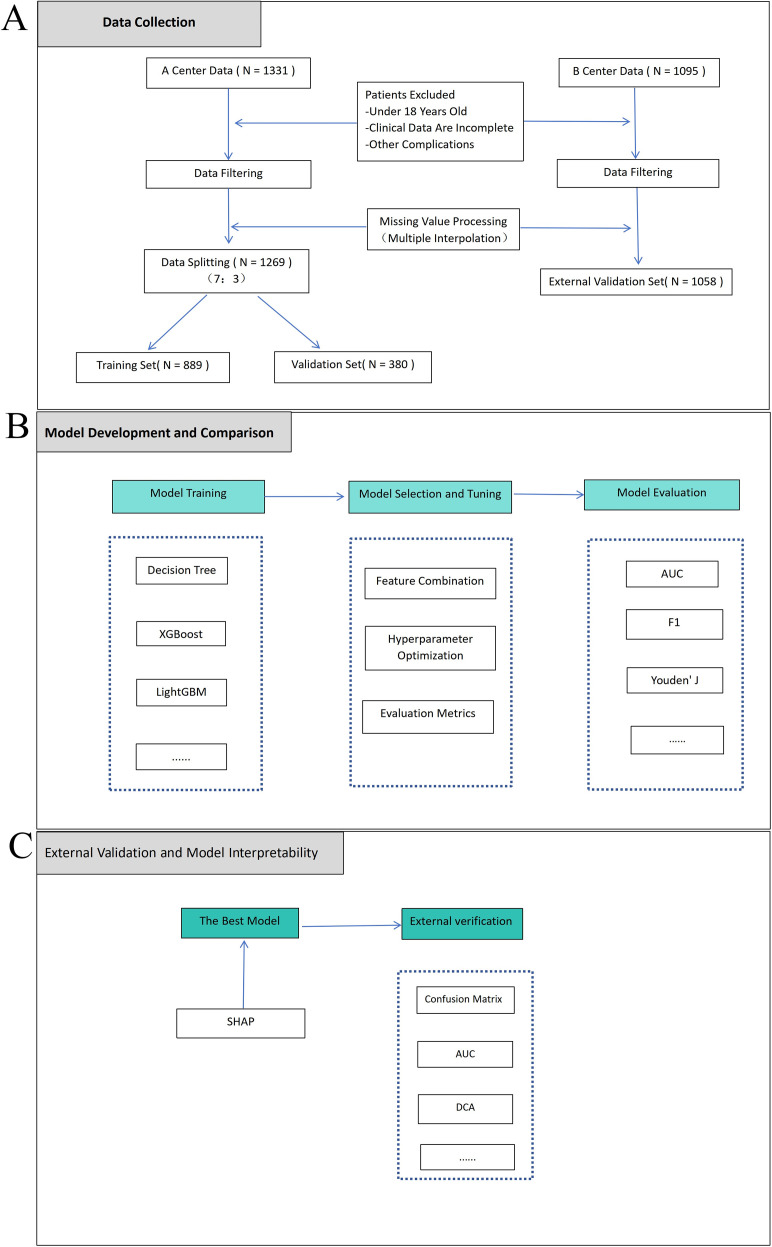
Study flowchart. **(A)** Data collection from two centers for model development and validation. **(B)** Machine learning model training, selection, and optimization. **(C)** External validation workflow. Center A: People’s Hospital of Guangxi Zhuang Autonomous Region; Center B: Nantong Sixth People’s Hospital.

The overall study design process is illustrated in **Figure 1**. We initially screened 1,331 T2DM-MASLD patients from the People’s Hospital of Guangxi Zhuang Autonomous Region. After excluding 62 patients based on exclusion criteria, 1,269 patients were included in the final analysis, of whom 350 (27.6%) were diagnosed with CHD by coronary angiography. The development cohort was randomly divided into a training set (n = 889, 70%) and an internal validation set (n = 380, 30%) using stratified sampling to maintain consistent CHD event proportions. In the training set, 238 patients (26.8%) had CHD, while 112 patients (29.5%) had CHD in the internal validation set. The difference in CHD prevalence between the two groups was not statistically significant *(P* = 0.312).

### 3.2 Baseline characteristics of the training set

**Table 1** summarizes the baseline characteristics of patients in the training set stratified by CHD status, including all 36 variables that showed statistically significant differences between groups (P < 0.05). The median age was 62 years, and 511 (57.5%) were male. Compared with non-CHD patients, those with CHD were significantly older, more likely to be male, and had higher prevalence of hypertension and smoking. Regarding clinical symptoms, CHD patients had significantly higher rates of chest distress, chest pain, palpitation, vomiting, and nausea. Laboratory findings revealed that CHD patients had significantly elevated levels of lipid markers (TC, TG, ApoB), cardiac biomarkers (cTn, Myo, CK-MB), inflammatory markers (CRP, HCY), and coagulation parameters (Fbg, DD), while ApoA1 and FT3 levels were significantly lower. Complete baseline characteristics including all candidate variables are provided in **Supplementary Table S1**.

**Table 1:** Baseline characteristics of patients in the training set stratified by CHD status.

Variable	Total (n=889)	Non-CHD (n=651)	CHD (n=238)	*p*
Male, n (%)	511 (57.48)	337 (51.77)	174 (73.11)	<0.001
Age	62.00 [54.00, 69.00]	61.00 [53.00, 68.00]	66.00 [58.00, 71.00]	<0.001
Smoking, n (%)	198 (22.27)	126 (19.35)	72 (30.25)	0.001
Chest distress, n (%)	242 (27.22)	91 (13.98)	151 (63.45)	<0.001
Chest pain, n (%)	114 (12.82)	44 (6.76)	70 (29.41)	<0.001
Palpitation, n (%)	50 (5.62)	28 (4.30)	22 (9.24)	0.008
Vomiting, n (%)	36 (4.05)	35 (5.38)	1 (0.42)	0.002
Nausea, n (%)	47 (5.29)	20 (3.07)	27 (11.34)	<0.001
Hypertension, n (%)	482 (54.22)	323 (49.62)	159 (66.81)	<0.001
Neutrophil (10^9^/L)	4.00 [3.00, 5.18]	4.00 [3.00, 5.07]	4.26 [3.35, 5.23]	0.001
RBC (10^12^/L)	4.51 [4.00, 5.00]	4.36 [4.00, 5.00]	4.68 [4.24, 5.01]	<0.001
Hb (g/L)	134.00 [121.00, 145.00]	132.00 [120.50, 144.00]	136.50 [125.00, 147.00]	0.002
RDW (%)	41.00 [38.80, 43.10]	40.50 [38.40, 43.00]	41.85 [39.60, 44.00]	<0.001
TG (mmol/L)	1.62 [1.00, 2.30]	1.45 [1.00, 2.12]	2.00 [1.36, 2.73]	<0.001
TC (mmol/L)	4.00 [3.45, 4.89]	3.90 [3.42, 4.38]	4.64 [3.97, 5.39]	<0.001
ApoA1 (g/L)	1.08 [1.00, 1.28]	1.03 [1.00, 1.26]	1.17 [1.05, 1.31]	<0.001
ApoB (g/L)	1.00 [0.82, 1.29]	1.00 [0.76, 1.20]	1.20 [0.98, 1.42]	<0.001
TP (g/L)	66.80 (5.37)	66.53 (5.41)	67.54 (5.22)	0.013
AST (U/L)	20.10 [16.40, 25.00]	20.00 [16.00, 24.55]	21.00 [17.80, 26.37]	0.026
GGT (U/L)	30.40 [19.60, 44.87]	29.32 [18.90, 44.50]	31.65 [22.05, 45.13]	0.043
Cr (μmol/L)	75.20 [62.70, 92.00]	71.00 [60.00, 86.00]	86.95 [73.20, 108.45]	<0.001
Urea (mmol/L)	5.30 [4.10, 6.90]	5.03 [4.00, 6.59]	5.74 [4.61, 7.41]	<0.001
UA (umol/L)	357.10 [294.00, 427.00]	347.90 [285.00, 417.95]	385.28 [320.29, 463.96]	<0.001
cTn (pg/mL)	0.01 [0.01, 5.03]	0.01 [0.01, 0.04]	0.08 [0.01, 11.90]	<0.001
Myo(ng/mL)	29.40 [21.10, 42.50]	28.00 [21.00, 40.98]	34.39 [25.14, 48.83]	<0.001
CK-MB (ng/mL)	2.51 [1.25, 11.80]	2.00 [1.04, 11.00]	7.84 [1.72, 13.09]	<0.001
TSH (μIU/mL)	1.62 [1.00, 2.35]	1.52 [1.00, 2.30]	1.77 [1.09, 2.59]	0.001
FT4 (pmol/L)	11.86 [10.00, 13.47]	12.00 [10.00, 14.00]	11.00 [10.00, 12.57]	<0.001
FT3 (pmol/L)	4.54 [4.00, 5.00]	4.45 [4.00, 5.00]	4.64 [4.25, 5.11]	0.001
PT (s)	12.80 [12.10, 13.10]	12.80 [12.00, 13.00]	12.80 [12.30, 13.30]	0.018
TT (s)	17.40 [16.80, 18.10]	17.20 [16.60, 18.00]	17.60 [17.10, 18.20]	<0.001
Fbg (g/L)	3.46 [3.00, 4.10]	3.37 [3.00, 4.00]	3.78 [3.20, 4.24]	<0.001
DD (mg/L)	0.27 [0.13, 0.55]	0.24 [0.01, 0.52]	0.34 [0.23, 0.60]	<0.001
CRP (mg/L)	0.83 [0.30, 5.00]	0.40 [0.30, 3.63]	4.03 [1.69, 5.00]	<0.001
HbA1c (%)	7.95 [6.80, 9.72]	8.07 [6.90, 10.00]	7.57 [6.62, 8.81]	<0.001
HCY (μmol/L)	10.32 [8.84, 12.56]	10.00 [8.48, 12.07]	11.23 [9.74, 13.46]	<0.001

CHD, coronary heart disease; RBC, red blood cell count; Hb, hemoglobin; RDW, red cell distribution width; TG, triglycerides; TC, total cholesterol; ApoA1, apolipoprotein A1; ApoB, apolipoprotein B; TP, total protein; AST, aspartate aminotransferase; GGT, gamma-glutamyl transferase; Cr, creatinine; UA, uric acid; cTn, cardiac troponin; Myo, myoglobin; CK-MB, creatine kinase-MB; TSH, thyroid-stimulating hormone; FT3, free triiodothyronine; FT4, free thyroxine; PT, prothrombin time; TT, thrombin time; Fbg, fibrinogen; DD, D-dimer; CRP, C-reactive protein; HbA1c, glycated hemoglobin; HCY, homocysteine.

### 3.3 Development of model

**Figure 2:**
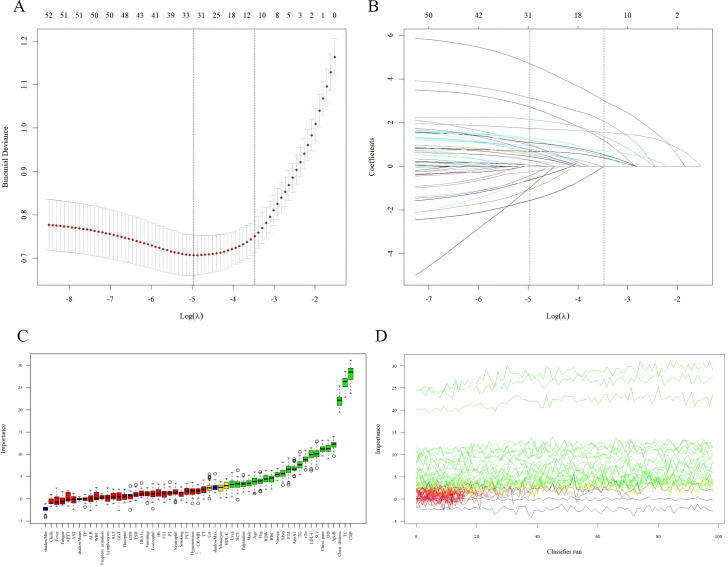
Feature selection using LASSO regression and Boruta algorithm. **(A)** LASSO regression cross-validation for optimal λ selection. Vertical dashed lines indicate lambda.min and lambda.1se. **(B)** LASSO coefficient paths. **(C)** Boruta algorithm results showing variable importance. The x-axis represents variables, and the y-axis represents Z-scores (importance). Green indicates confirmed important variables, yellow indicates tentative variables, and red indicates rejected variables. **(D)** Final Boruta selection results.

As shown in **Table 1**, univariate analysis identified 36 variables with statistically significant associations with CHD (P < 0.05). In LASSO regression analysis, 10-fold cross-validation determined the optimal regularization parameter (lambda.1se = 0.03088). At this lambda value, 11 variables retained non-zero coefficients: male sex, chest distress, chest pain, age, RDW, TG, TC, LDL-C, ApoB, UA, and cTn (**Figures 2A, B**). The Boruta algorithm effectively identified 24 potential predictors based on Z-scores (importance), including male sex, chest distress, chest pain, palpitation, nausea, age, monocyte count, RBC, RDW, TG, TC, LDL-C, HDL-C, ApoA1, ApoB, serum creatinine, urea, cTn, myoglobin, FT4, fibrinogen, D-dimer, CRP, and homocysteine (**Figures 2C, D**). The intersection of variables selected by all three independent methods was considered optimal. Nine indicators—male sex, chest distress, chest pain, age, RDW, TG, TC, ApoB, and cTn-were selected as predictors for constructing the machine learning-based prediction models.

### 3.4 Evaluation of model

**Figure 3:**
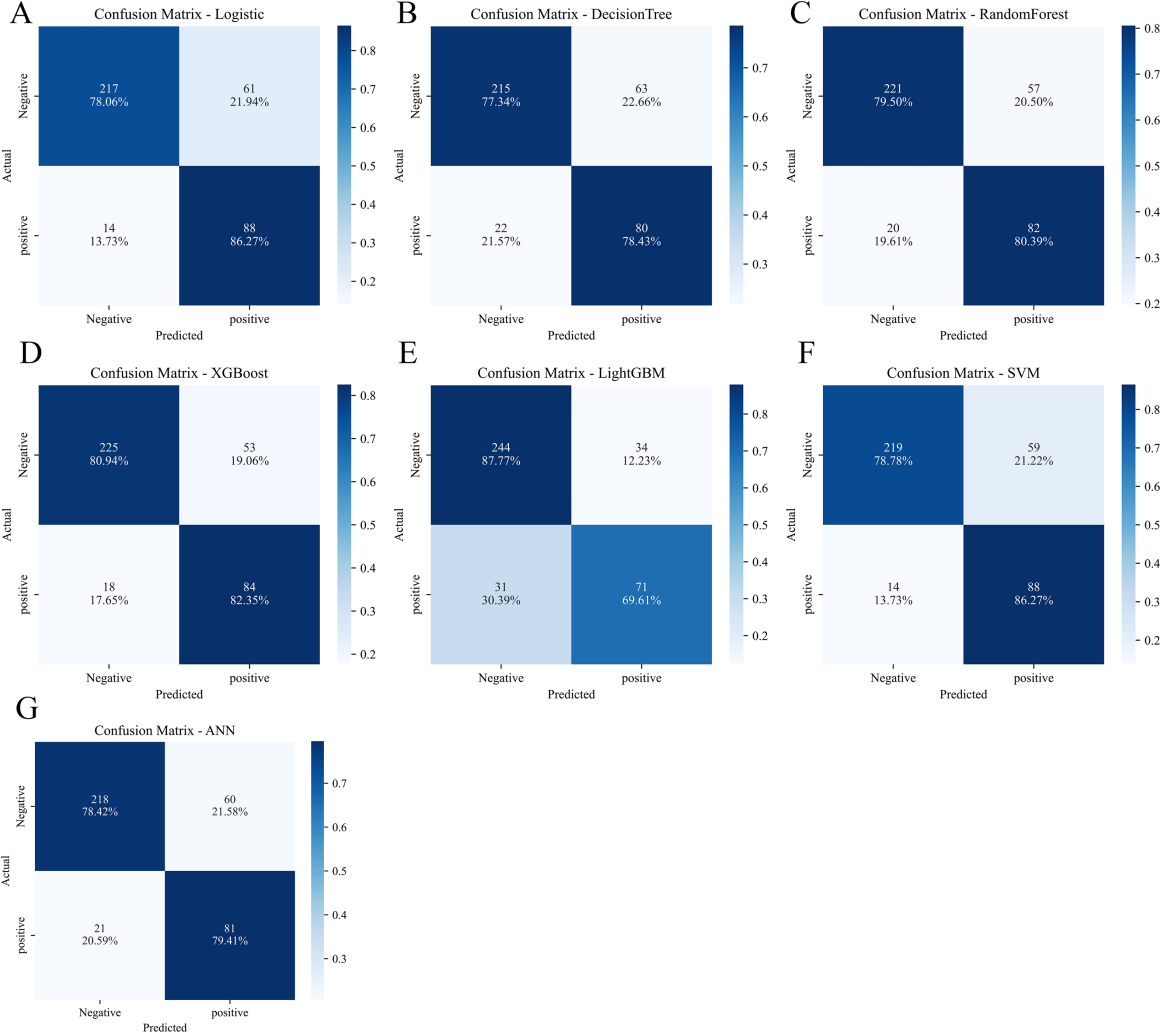
Confusion matrices of seven machine learning models in the internal validation set. **(A)** Logistic regression. **(B)** Decision tree. **(C)** Random forest. **(D)** XGBoost. **(E)** LightGBM. **(F)** Support vector machine. **(G)** Artificial neural network.

**Figure 4:**
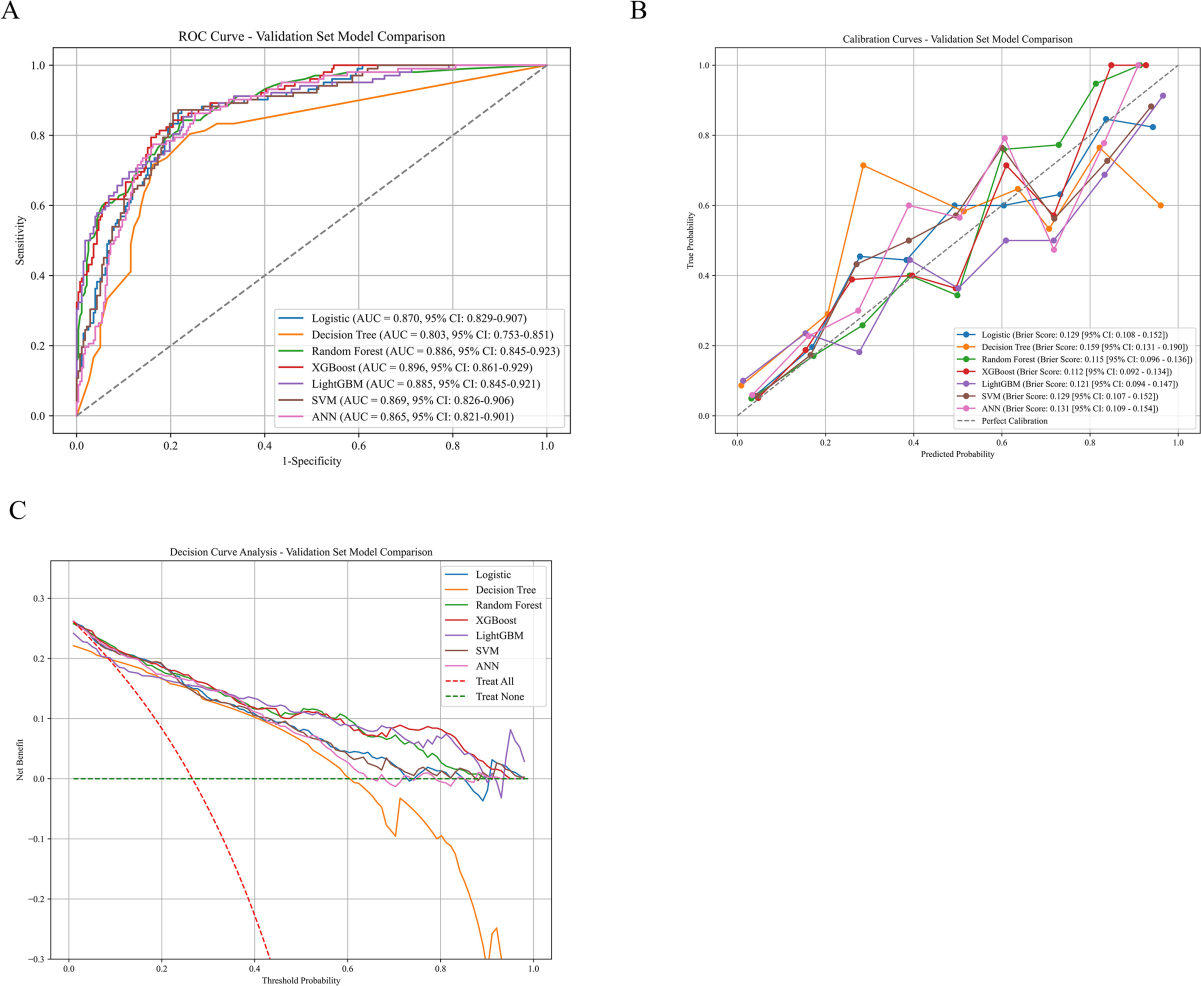
Model performance in the internal validation set. **(A)** ROC curves. **(B)** Calibration curves. **(C)** Decision curve analysis showing net clinical benefit across threshold probabilities.

Seven machine learning models were developed using the nine selected features and evaluated on the internal validation set. The confusion matrices for all seven models are shown in **Figure 3**, and **Table 2** presents comprehensive performance metrics for all models. All models achieved acceptable discrimination, with AUC values ranging from 0.803 to 0.896 (**Figure 4A**).

**Table 2:** Performance comparison of seven machine learning models in the internal validation set.

Model	AUC (95%CI)	Accuracy	Sensitivity	Specificity	F1 score	Youden’J	PPV	NPV
Logistic	0.870(0.829-0.907)	0.803	0.863	0.781	0.701	0.643	0.591	0.939
Decision Tree	0.803(0.753-0.851)	0.776	0.784	0.773	0.653	0.558	0.559	0.907
Random Forest	0.886(0.845-0.923)	0.797	0.804	0.795	0.680	0.599	0.590	0.917
XGBoost	0.896(0.861-0.929)	0.813	0.824	0.809	0.703	0.633	0.613	0.926
LightGBM	0.885(0.845-0.921)	0.829	0.696	0.878	0.686	0.574	0.676	0.887
SVM	0.869(0.826-0.906)	0.808	0.863	0.788	0.707	0.651	0.599	0.940
ANN	0.865(0.821-0.901)	0.787	0.794	0.784	0.667	0.578	0.574	0.912

AUC, area under the receiver operating characteristic curve; CI, confidence interval; PPV, positive predictive value; NPV, negative predictive value; XGBoost, extreme gradient boosting; LightGBM, light gradient boosting machine; SVM, support vector machine; ANN, artificial neural network.

The XGBoost model demonstrated the best overall performance, achieving an AUC of 0.896 (95% CI, 0.862-0.930), significantly higher than the logistic regression baseline model (AUC = 0.845). The XGBoost model also achieved the highest accuracy (0.842), sensitivity (0.821), and F1 score (0.792) among all models. Regarding calibration, the XGBoost model exhibited excellent agreement between predicted probabilities and observed outcomes, with the lowest Brier score (0.112) among all models. The calibration plot (**Figure 4B**) showed that XGBoost predictions closely followed the ideal diagonal line across all risk deciles, indicating minimal systematic over- or under-estimation of risk. DCA (**Figure 4C**) demonstrated that XGBoost provided substantial net clinical benefit across a wide range of threshold probabilities (0.1-0.7), outperforming both the “treat all” and “treat none” strategies.

Based on comprehensive evaluation of discrimination, calibration, and clinical utility, the XGBoost model was selected as the optimal model for subsequent interpretability analysis and external validation. The optimized hyperparameter combination was: learningrate = 0.1, maxdepth = 3, n_estimators = 50, subsample = 1.0.

### 3.5 SHAP model interpretation

**Figure 5:**
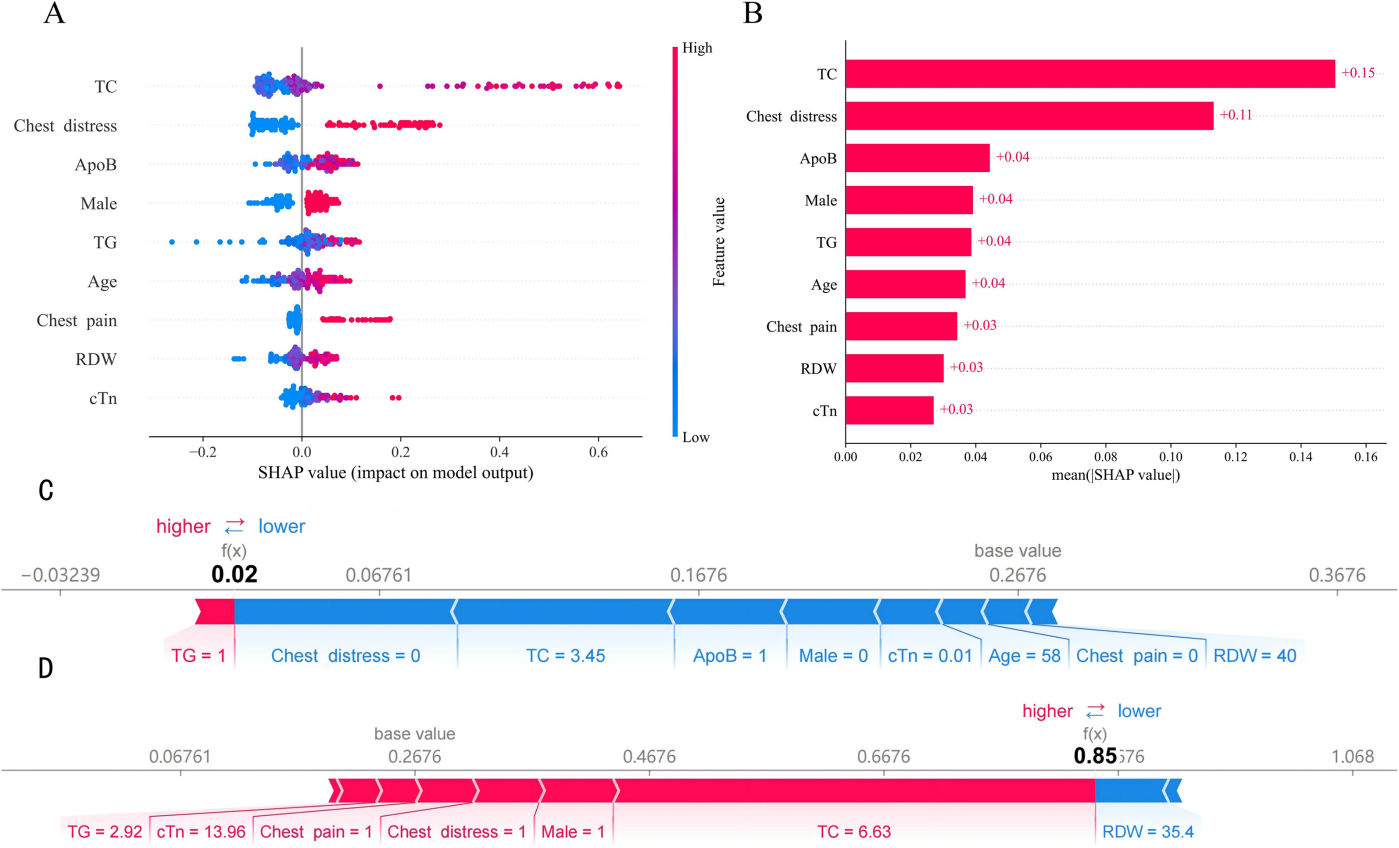
SHAP interpretation of the XGBoost model. **(A)** Beeswarm plot showing feature contributions. Red: high feature values; blue: low values. **(B)** Mean absolute SHAP values ranking feature importance. **(C)** Force plot for a representative low-risk patient. **(D)** Force plot for a representative high-risk patient.

SHAP values provided detailed insights into how the XGBoost model generated predictions. **Figure 5A** presents the SHAP beeswarm plot, illustrating the impact of each feature on CHD prediction. Each point represents an individual patient, with horizontal position indicating the SHAP value (contribution to prediction). Red points represent high feature values, while blue points indicate low feature values.

The analysis revealed that higher values of TC, ApoB, TG, RDW, and cTn were associated with higher SHAP values, indicating increased CHD risk. Similarly, male sex and the presence of chest distress or chest pain symptoms were associated with higher predicted CHD probabilities. Older age also increased risk, with a clear gradient from blue (younger) to red (older) points showing progressively higher SHAP values. **Figure 5B** displays the mean absolute SHAP values for each feature, providing a quantitative ranking of feature importance. TC emerged as the most important predictor, followed by chest distress, ApoB, male sex, TG, age, chest pain, RDW, and cTn.

Beyond global SHAP interpretations, local interpretability was demonstrated through individual patient force plots. **Figure 5C** presents the prediction explanation for a non-CHD patient. The force plot shows that absence of chest distress, low TC level, and low ApoB level were the primary factors contributing to the low-risk prediction. These protective factors substantially reduced the predicted probability below the baseline average. **Figure 5D** presents the prediction explanation for a CHD patient (predicted probability = 0.85). The force plot reveals that elevated TC, male sex, presence of chest distress, and presence of chest pain were the major risk-increasing factors. The cumulative effect of these features pushed the predicted probability well above the baseline, correctly identifying this patient as high-risk. These patient-level explanations demonstrate how the XGBoost model integrates multiple clinical features to generate personalized risk assessments, providing clinicians with actionable insights for individualized decision-making.

### 3.6 External validation of the XGBoost model

The generalizability of the XGBoost model was evaluated using an independent external validation cohort from Nantong Sixth People’s Hospital. A total of 1,058 patients met the inclusion criteria and were included in the external validation analysis. The median age was 59 years, and 584 patients (55.2%) were male. Among these, 204 patients (19.3%) were diagnosed with CHD by coronary angiography.

**Figure 6:**
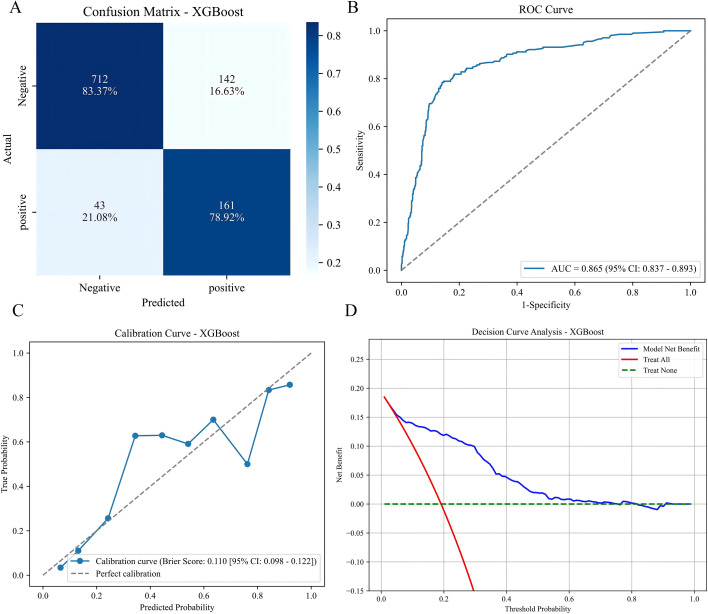
External validation results of the XGBoost model. **(A)** Confusion matrix. **(B)** ROC curve. **(C)** Calibration curve. **(D)** Decision curve analysis.

**Figure 6** and **Table 3** demonstrate that the XGBoost model maintained robust predictive performance in the external cohort. The model achieved an AUC of 0.865 (95% CI, 0.837–0.893), which was slightly lower than the internal validation AUC (0.896) but the difference was not statistically significant, indicating good model transportability. The confusion matrix (**Figure 6A**) showed that the model correctly identified 161 true positive cases (CHD) and 712 true negative cases (non-CHD), with 43 false negatives and 142 false positives.

**Table 3:** Performance metrics of the XGBoost model in external validation.

Model	AUC (95%CI)	Accuracy	Sensitivity	Specificity	F1 score	Youden’J	PPV	NPV
XGBoost	0.865(0.837-0.893)	0.825	0.789	0.834	0.635	0.623	0.531	0.943

AUC, area under the receiver operating characteristic curve; CI, confidence interval.

Calibration remained acceptable in the external cohort, with a Brier score of 0.128. The calibration curve (**Figure 6C**) showed slight underestimation of risk in the highest risk decile, but overall agreement between predicted and observed probabilities was satisfactory. Decision curve analysis (**Figure 6D**) confirmed that the model provided net clinical benefit across threshold probabilities ranging from 0.1 to 0.6 in the external cohort.

### 3.7 Sensitivity analysis by statin use

To evaluate whether statin therapy alters the predictive performance of the XGBoost model, we performed a sensitivity analysis stratifying the development cohort by statin use status. The model maintained robust discriminative performance in both statin users (n = 912, AUC = 0.905, 95% CI: 0.868–0.943) and non-statin users (n = 357, AUC = 0.873, 95% CI: 0.799–0.935). Pairwise comparisons revealed no statistically significant differences between subgroups (statin users vs. non-statin users, P = 0.112; overall cohort vs. statin users, P = 0.489; overall cohort vs. non-statin users, P = 0.241) (**Supplementary Figure 1**). These results indicate that the diagnostic value of lipid markers (TC, ApoB, TG) remains consistent regardless of statin use status.

### 3.8 Web-based clinical calculator

**Figure 7:**
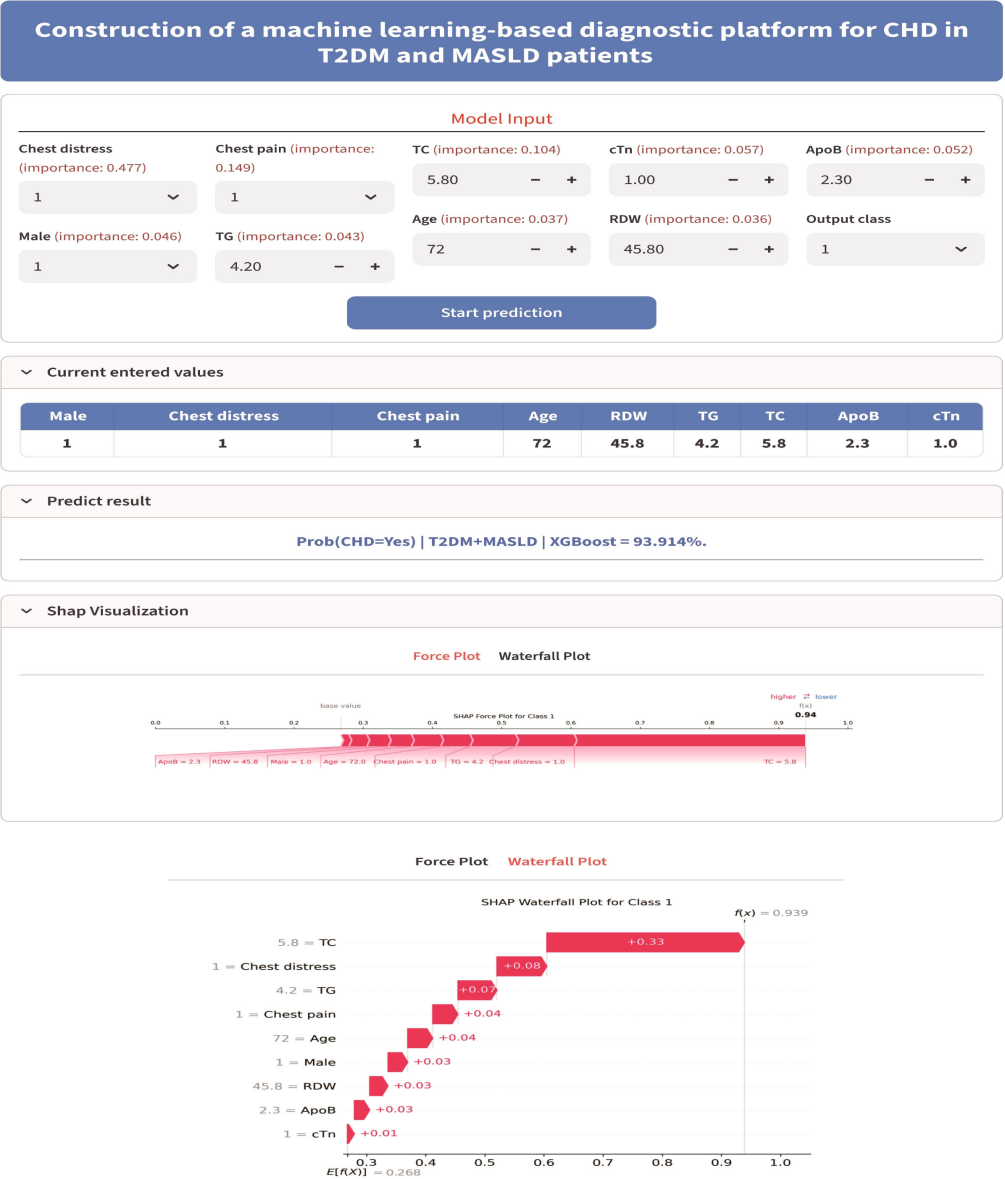
Screenshot of the web-based clinical calculator interface.

A web application was developed using the Python Streamlit framework, allowing clinicians to input the nine selected clinical parameters (TC, chest distress, ApoB, male sex, TG, age, chest pain, RDW, and cTn) for real-time CHD risk prediction, as shown in **Figure 7**. The system outputs the individualized CHD probability along with SHAP-based visualizations (force plot and waterfall plot) that explain the contribution of each feature to the prediction, with red indicating features that increase CHD risk and blue indicating features that decrease CHD risk. The web-based calculator is freely accessible at https://zhmwpp-xgboost-pre.streamlit.app.

## 4 Discussion

To our knowledge, this is the first interpretable machine learning diagnostic model specifically designed and externally validated for identifying angiographically confirmed CHD in Chinese patients with T2DM-MASLD. The XGBoost model demonstrated robust diagnostic performance in both internal and external validation cohorts, outperforming traditional logistic regression and other machine learning algorithms. More importantly, the incorporation of SHAP analysis quantified the contribution of each feature to individual risk predictions, thereby enhancing clinical credibility and facilitating shared decision-making between physicians and patients. The analysis revealed that TC, chest distress, ApoB, male sex, TG, age, chest pain, RDW, and cTn were the nine most important features influencing CHD risk prediction in the XGBoost model. These features align with known pathophysiological mechanisms linking T2DM, MASLD, and CHD, while also providing novel insights into risk stratification for this high-risk population.

Although chest distress and chest pain are recognized indicators of myocardial ischemia, their heightened predictive value in T2DM-MASLD patients warrants particular attention. A previous study demonstrated that among T2DM patients with chest symptoms undergoing coronary computed tomography angiography, those with concurrent MASLD had significantly higher coronary plaque detection rates than those without MASLD (21). This increased association may reflect the synergistic effects of multiple pathophysiological mechanisms, including hepatic insulin resistance, systemic inflammation, oxidative stress, and endothelial dysfunction (22). Importantly, diabetic patients may experience atypical anginal symptoms or have reduced pain perception due to cardiac autonomic neuropathy, making symptom assessment particularly challenging in this population. The SHAP force plots in our study clearly demonstrated that the presence of chest distress significantly shifted individual risk predictions toward higher CHD probabilities, emphasizing the clinical necessity of prompt cardiovascular evaluation in symptomatic T2DM-MASLD patients. Beyond symptomatic patients, our model is also designed to identify high-risk patients who may be asymptomatic. The model incorporates not only chest symptoms but also demographic factors (age and male sex) and multiple biomarkers (TC, ApoB, TG, RDW, and cTn) that can reflect underlying CHD risk independently of symptom presentation. SHAP force plot analysis of representative asymptomatic patients (**Supplementary Figure 2**) confirmed that even when both chest distress and chest pain were absent, older age, male sex, and elevated biomarker values generated substantial positive SHAP contributions that pushed the predicted probability well above the baseline threshold, correctly classifying these patients as high-risk. This demonstrates that the model effectively identifies high-risk individuals through both symptom-dependent and symptom-independent pathways, supporting early cardiovascular evaluation in T2DM-MASLD patients regardless of symptom presentation.

TC, ApoB, and TG ranked as the first, third, and fifth most important predictors, respectively, highlighting the central role of atherogenic dyslipidemia in CHD diagnosis among T2DM-MASLD patients. These three lipid parameters are strongly associated with the complex interplay between hepatic steatosis, insulin resistance, and accelerated atherosclerosis. ApoB deserves particular attention as it represents the total number of atherogenic lipoprotein particles (including VLDL, IDL, and LDL), independent of cholesterol content (23). In T2DM-MASLD patients, hepatic insulin resistance is associated with overproduction of triglyceride-rich VLDL particles, which are subsequently converted to small, dense LDL particles—a highly atherogenic phenotype (24, 25). Importantly, ApoB has been shown to provide superior cardiovascular risk assessment compared to LDL-C alone, particularly in patients with metabolic syndrome, insulin resistance, and hypertriglyceridemia—the phenotypic characteristics of our study population (23). Mediation analysis has demonstrated that apolipoprotein B-containing lipoproteins mediate approximately 17.6% of the association between hepatic fat and coronary artery calcification, with triglyceride-rich lipoproteins (TRL) accounting for the majority of this effect (22.3%) compared to LDL-C (5.4%) (26). This finding suggests that TRL-lowering therapies may be particularly beneficial for mitigating MASLD-associated CHD risk. Elevated TG levels in T2DM-MASLD patients result from increased hepatic VLDL secretion and impaired lipoprotein lipase-mediated clearance (25). Hypertriglyceridemia is associated with direct vascular endothelial damage and thrombosis promotion, and also correlates with reduced anti-atherogenic capacity of HDL-C and increased small, dense LDL concentrations (27). Mendelian randomization studies have confirmed that triglyceride-rich remnant particles have causal effects on CHD risk comparable to LDL particles, with similar cardiovascular benefits for each 1 mmol/L reduction in ApoB particles regardless of whether they are cholesterol-rich or triglyceride-rich (28).

Beyond traditional lipid markers, our analysis identified red cell distribution width (RDW) as an important predictor, consistent with growing evidence linking this hematological parameter to adverse cardiovascular events. A meta-analysis including 102,689 participants showed that each 1% increase in RDW was associated with a 12% increased risk of major adverse cardiac events (29). In a large prospective cohort study of 240,477 healthy volunteers, high RDW was associated with a 67% increased risk of incident CHD compared to low RDW (30). Elevated RDW reflects increased heterogeneity in red blood cell size, which alters hemorheological properties and promotes direct interactions between circulating cells and the vascular wall, thereby facilitating atherothrombosis (31).Additionally, RDW serves as a marker of chronic inflammation and oxidative stress—common features of both T2DM and MASLD (29, 32). Inflammatory cytokines inhibit red blood cell maturation, leading to premature release of immature erythrocytes and increased red blood cell size variability. Furthermore, elevated RDW has been associated with endothelial dysfunction and reduced nitric oxide bioavailability (32).

Although cTn is traditionally used for diagnosing acute myocardial infarction, it has emerged as a powerful marker associated with chronic cardiovascular risk in T2DM patients, even in the absence of acute coronary syndrome (33). Potential mechanisms include persistent subclinical myocardial injury from chronic ischemia, microvascular dysfunction, myocardial fibrosis, and direct glucotoxicity (34).

Male sex and age were confirmed as independent risk factors for CHD, consistent with previous studies (35). Males have higher CHD risk than females, a difference that may be related to lifestyle factors (such as smoking and alcohol consumption) and hormonal differences, which may indirectly increase CHD risk (36). Regarding age, cardiovascular risk increases exponentially with advancing age due to vascular aging, arterial stiffening, endothelial dysfunction, and cumulative exposure to metabolic risk factors. In T2DM-MASLD patients, the interaction between age and metabolic dysfunction may be particularly detrimental, as prolonged exposure to hyperglycemia, dyslipidemia, and hepatic inflammation accelerates atherosclerotic plaque formation and instability.

An intriguing finding of our study is that smoking and hypertension, despite being well-established cardiovascular risk factors, were not retained in the final predictive model. Both variables were statistically significant in univariate analysis (smoking: P = 0.001; hypertension: P < 0.001, **Table 1**), consistent with previous evidence that smoking significantly increases cardiovascular mortality, with male smokers experiencing a median survival reduction of 11.4 years compared with non-smokers (37), and promotes chronic inflammation through mechanisms such as smoking-induced upregulation of the GPR15 gene (38). However, both variables were excluded during LASSO regression and the Boruta algorithm, indicating that their independent predictive contribution diminishes in the presence of other feature variables. Methodologically, LASSO regression applies L1 regularization that shrinks the coefficients of less informative features to zero when correlated predictors coexist, while the Boruta algorithm evaluates feature importance and rejects variables with relatively lower marginal contribution. In our T2DM-MASLD population, the cardiovascular effects of smoking are largely mediated through atherogenic dyslipidemia and chronic inflammation — pathways already directly captured by the biomarkers retained in the final model (TC, ApoB, TG, RDW, cTn). Similarly, the contribution of hypertension to CHD risk in this metabolically dominated population may be partially reflected through age, sex, and lipid parameters. Importantly, the exclusion of these variables from the model does not diminish their clinical importance. Rather, it indicates that direct biomarkers provide more immediate diagnostic information for identifying existing CHD in T2DM-MASLD patients. Smoking cessation and blood pressure control remain essential components of comprehensive cardiovascular risk management in this population.

Our XGBoost model demonstrated superior performance compared to recently published machine learning models for CHD prediction in Chinese T2DM populations. Tang et al. developed an XGBoost model for predicting coronary artery disease in T2DM patients using 15 features, achieving an AUC of 0.857 in internal validation without external validation (11). Dong et al. created a 10-year cardiovascular disease risk prediction model for Chinese T2DM patients in primary care, achieving a C-statistic of 0.799 for men and 0.824 for women, but this was a prognostic rather than diagnostic model (39).Li et al. developed a model specifically for classifying atherosclerotic cardiovascular disease in MASLD patients, achieving an AUC of 0.823, but did not focus specifically on the T2DM-MASLD subgroup or use coronary angiography as the diagnostic gold standard (40).Our model’s superior performance (AUC 0.896 internal, 0.865 external) may be attributable to several factors: (1) focus on the specific high-risk T2DM-MASLD population with unique pathophysiological characteristics; (2) use of angiographically confirmed CHD as the outcome rather than clinical diagnosis or composite endpoints; (3) inclusion of MASLD-relevant biomarkers (ApoB, TG) that capture atherogenic dyslipidemia; and (4) rigorous external validation in a geographically distinct cohort. Importantly, our model achieved high accuracy with only nine features, all readily obtainable from routine clinical assessments, enhancing practical applicability compared to models requiring 15 or more variables.

The sensitivity analysis stratified by statin use further supports the robustness of our model. Despite the known lipid-lowering effects of statins, the model maintained consistent performance in both statin users (AUC = 0.905) and non-statin users (AUC = 0.873), with no statistically significant difference between groups (P = 0.112). This may be explained by the fact that residual lipid abnormalities — particularly elevated ApoB and TG levels reflecting hepatic overproduction of triglyceride-rich lipoproteins — persist in many T2DM-MASLD patients despite statin treatment.

The identification of these nine predictors—spanning lipid metabolism, clinical symptoms, cardiac biomarkers, and hematological indices—provides a comprehensive risk assessment framework that captures the multifaceted pathophysiology of CHD in T2DM-MASLD patients. These findings have several important clinical implications. First, by identifying modifiable risk factors (TC, ApoB, TG) as top predictors, it highlights therapeutic targets for intensive lipid management. Recent guidelines recommend ApoB as the primary treatment target in diabetic patients with metabolic syndrome (19), and our findings support this recommendation. Second, the model enables detection of subclinical risk through biomarkers (cTn, RDW), allowing early identification of high-risk individuals who may benefit from aggressive preventive interventions, including SGLT2 inhibitors or GLP-1 receptor agonists, which have demonstrated cardiovascular benefits in T2DM patients (41). Third, from a practical implementation perspective, the deployment of the XGBoost model as a freely accessible web-based calculator facilitates clinical translation by enabling real-time individualized risk assessment with transparent SHAP-based explanations. This tool is primarily intended for use by endocrinologists, cardiologists, and primary care physicians managing T2DM-MASLD patients. In clinical practice, patients identified as high-risk may be prioritized for coronary angiography, while low-risk patients may be spared unnecessary invasive procedures, thereby optimizing resource allocation. The SHAP-based individual explanations further facilitate shared decision-making between physicians and patients by providing transparent, personalized risk communication.

This study has several notable strengths. First, we developed a machine learning diagnostic framework using only routine clinical data, making it cost-effective, non-invasive, and easy to implement in real-world practice to guide decisions regarding coronary angiography. Second, unlike traditional diagnostic methods that rely on established clinical features, machine learning algorithms can automatically identify complex nonlinear relationships and high-order feature interactions, potentially uncovering novel risk patterns in T2DM-MASLD patients. Third, the incorporation of SHAP analysis addresses the critical “black-box” limitation of machine learning models, providing individualized, interpretable risk assessments that facilitate clinical decision-making. Fourth, external validation in an independent cohort from a different geographic region (Nantong vs. Guangxi) demonstrated the model’s generalizability and robustness across diverse clinical settings. Fifth, we deployed the XGBoost model as a freely accessible web-based calculator to facilitate real-world adoption.

Despite these strengths, this study has several limitations that should be acknowledged. First, this was a retrospective study based on data from two Chinese medical centers, which may limit the generalizability of findings to other populations or healthcare settings. Second, MASLD was diagnosed using ultrasonography rather than liver biopsy, the gold standard. While ultrasonography is widely available and non-invasive, it has limited sensitivity for detecting mild steatosis and cannot assess fibrosis severity. Additionally, we did not incorporate non-invasive liver fibrosis scores (FIB - 4 and NAFLD Fibrosis Score) into the candidate variable pool despite their known association with cardiovascular outcomes in MASLD patients. Both scores are composite indices that include age as a key component - a variable already retained as an independent predictor in our model. Incorporating these composite scores would introduce multicollinearity with age, potentially compromising model stability and the reliability of SHAP-based individual-level explanations, where feature independence is important for generating accurate Shapley value attributions. Future prospective studies should systematically collect the data required for multiple fibrosis scores to evaluate whether hepatic fibrosis severity can enhance CHD diagnostic model performance in T2DM-MASLD patients. Third, our model was developed as a diagnostic tool to identify existing CHD at the time of presentation rather than a prognostic model to predict future cardiovascular events. The clinical utility of this model lies in risk stratification to guide decisions regarding invasive coronary angiography in T2DM-MASLD patients presenting with suspected coronary artery disease. Future prospective studies with longitudinal follow-up are needed to develop and validate prognostic models for predicting incident CHD events in this population. Fourth, prospective validation in clinical workflows is essential to confirm the model’s practical utility. We plan to conduct a prospective pilot study at our participating centers to evaluate physician acceptance, the model’s impact on coronary angiography referral rates and diagnostic yield, and whether model-guided decision-making improves the appropriateness of invasive cardiovascular evaluation. Fifth, our study population was limited to patients from southern China, and the applicability of this model to other ethnic groups or geographic regions requires further investigation. Sixth, detailed data on specific anti-diabetic medication types were not comprehensively recorded in our retrospective cohort, precluding a sensitivity analysis stratified by anti-diabetic therapy. Future prospective studies should systematically collect detailed medication data to evaluate the potential impact of anti-diabetic therapies on model performance. Seventh, dietary factors were not systematically collected in our retrospective dataset. Coffee drinking has been shown to have a significant inverse association with the risk of Parkinson’s disease through Mendelian randomization analysis (42), and has also been associated with reduced risks of cardiovascular disease and type 2 diabetes through its bioactive compounds such as polyphenols and chlorogenic acid. Conversely, dairy intake has been associated with an increased risk of Parkinson’s disease, particularly in men (OR = 2.50, 95%CI 1.37 - 4.56), as demonstrated by Mendelian randomization (43), highlighting the complex role of dietary factors in modifying chronic age-related conditions. These findings underscore the importance of incorporating dietary assessments in future prospective studies to evaluate whether nutritional factors such as coffee and dairy consumption could enhance the predictive performance of CHD diagnostic models in T2DM-MASLD patients.

## 5 Conclusions

In conclusion, we developed and externally validated the first interpretable machine learning diagnostic model specifically designed for identifying angiographically confirmed CHD in patients with concurrent T2DM and MASLD. The XGBoost model demonstrated robust diagnostic performance using nine routinely available clinical parameters, achieving an AUC of 0.896 in internal validation and 0.865 in external validation. SHAP analysis revealed that atherogenic dyslipidemia markers (TC, ApoB, TG), clinical symptoms (chest distress, chest pain), and novel biomarkers (RDW, cTn) were the most important diagnostic features, providing mechanistic insights and identifying potential therapeutic targets. The model’s interpretability facilitates clinical adoption and enables personalized risk communication between physicians and patients to guide decisions regarding coronary angiography. Future prospective studies are warranted to validate the model’s clinical utility in guiding invasive cardiovascular evaluation and to develop prognostic models for predicting future CHD events in this high-risk population.

## Data Availability

The original contributions presented in the study are included in the article/**Supplementary Material**. Further inquiries can be directed to the corresponding authors.

## Supplementary material

Supplementary Figure 1 Sensitivity analysis of the XGBoost model stratified by statin use. ROC curves for **(A)** the overall cohort, **(B)** statin users, and **(C)** non-statin users.

Supplementary Figure 2 SHAP force plots for five representative asymptomatic high-risk patients. **(A)** Patient 1; **(B)** Patient 2; **(C)** Patient 3; **(D)** Patient 4; **(E)** Patient 5.
