# Supplementary figures and images for: Development and external validation of an interpretable machine learning model for diagnosing coronary heart disease in patients with type 2 diabetes and MASLD
Source: Front Endocrinol (Lausanne). 2026 May 15;17:1830594. doi: 10.3389/fendo.2026.1830594 (PMC13218859; doi:10.3389/fendo.2026.1830594)

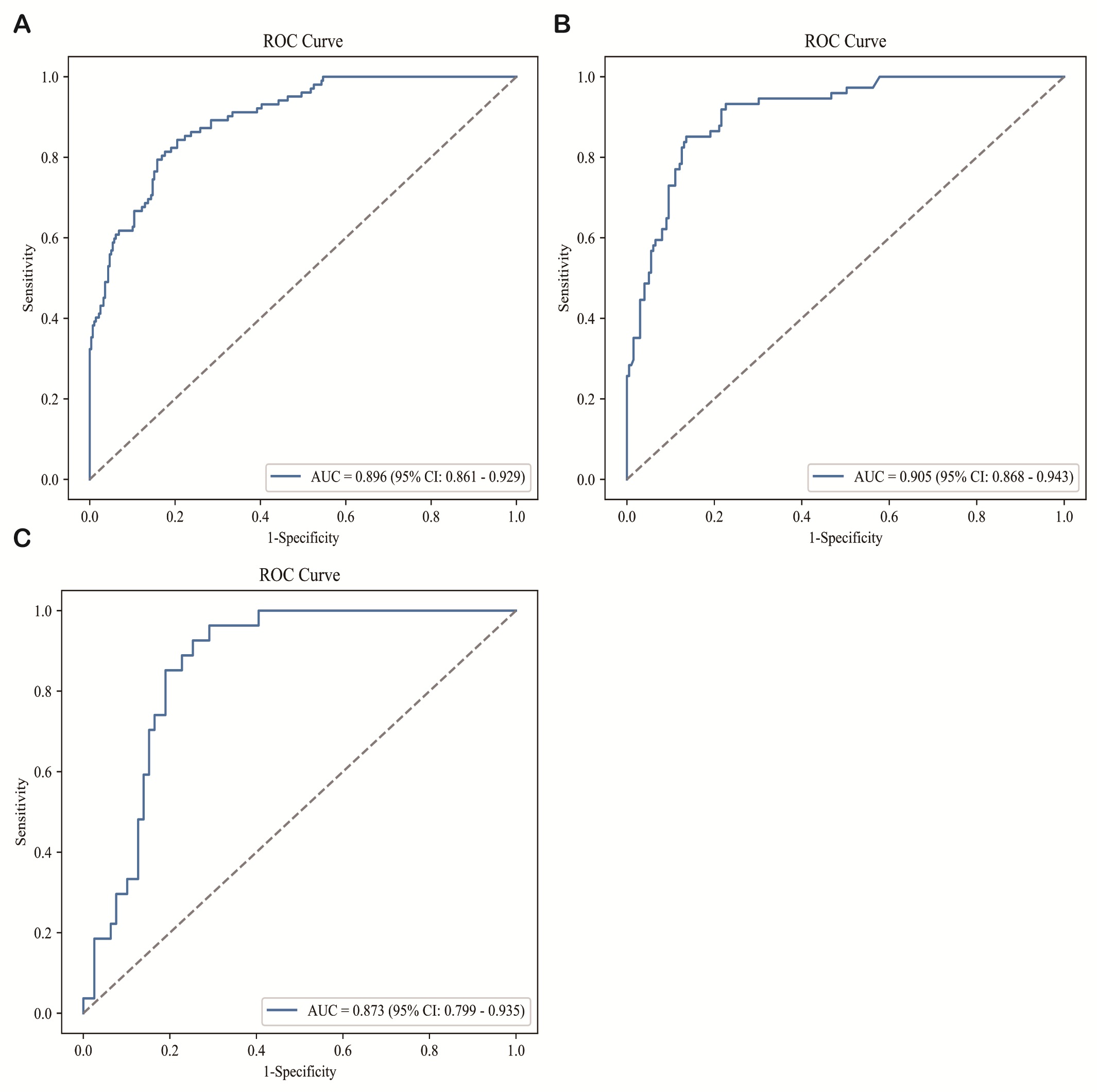

Supplement: Supplementary Figure 1 — Sensitivity analysis of the XGBoost model stratified by statin use. ROC curves for (A) the overall cohort, (B) statin users, and (C) non-statin users. [file Image1.jpeg]

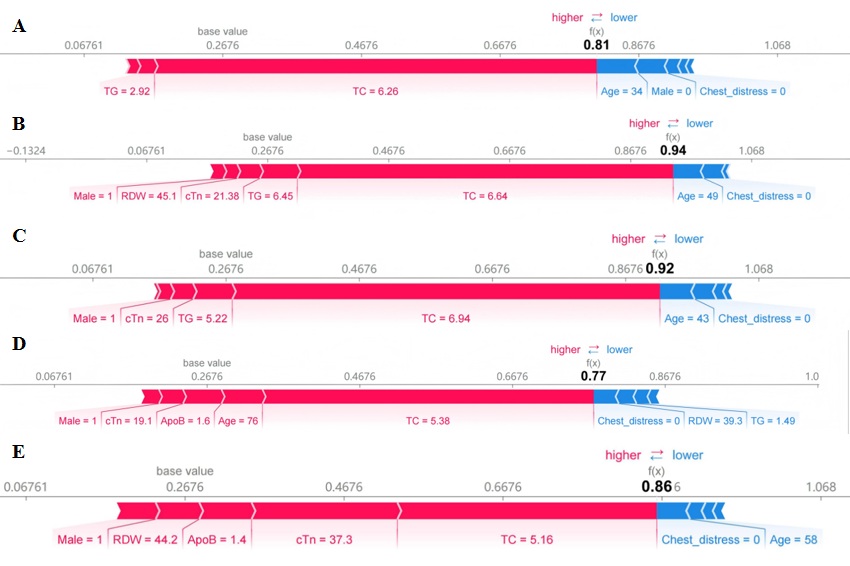

Supplement: Supplementary Figure 2 — SHAP force plots for five representative asymptomatic high-risk patients. (A) Patient 1; (B) Patient 2; (C) Patient 3; (D) Patient 4; (E) Patient 5. [file Image2.jpeg]
