# Supplementary material for: Development and external validation of an interpretable machine learning model for diagnosing coronary heart disease in patients with type 2 diabetes and MASLD
Source: Front Endocrinol (Lausanne). 2026 May 15;17:1830594. doi: 10.3389/fendo.2026.1830594 (PMC13218859; doi:10.3389/fendo.2026.1830594)
Supplement: Supplementary file 3 [file Table1.docx]

**Supplementary Material:**Development and External Validation of an Interpretable Machine Learning Model for Diagnosing Coronary Heart Disease in Patients with Type 2 Diabetes and MASLD

Chunxia Deng ^1†^, Lin Feng ^1†^, Tingting Li^2^, Suosu Wei ^3*^, Huiming Zhu^4*^, Jie Lu^1*^

^1^ Department of Endocrinology and Metabolism, Guangxi Academy of Medical Sciences and the People's Hospital of Guangxi Zhuang Autonomous Region, Nanning, Guangxi, China

^2^ Department of Respiratory and Critical Care Medicine, Guangxi Hospital of the First Affiliated Hospital, Sun Yat-sen University, Nanning, Guangxi, China

^3^ Clinical Research Center of Guangxi Academy of Medical Sciences, The People's Hospital of Guangxi Zhuang Autonomous Region, Nanning, Guangxi, China

^4^ Department of Clinical Laboratory, Nantong Sixth People's Hospital Affiliated to Shanghai University, Nantong, Jiangsu, 226011, China

†These authors have contributed equally to this work and share first authorship

*** Correspondence:**Suosu Wei
[sswei@gxams.org.cn](mailto:sswei@gxams.org.cn)

Huiming Zhu
[zhmwpp@163.com](mailto:zhmwpp@163.com)

Jie Lu
lujie16389@163.com

**Supplementary Table S1. Complete baseline characteristics of all candidate variables in the training set stratified by CHD status.**

| **Variable** | **Overall（n=889）** | **Non-CHD（n=651）** | **CHD（n=238）** | **p** |
| --- | --- | --- | --- | --- |
| Male, n (%) | 511 (57.48) | 337 (51.77) | 174 (73.11) | <0.001 |
| Age | 62.00 [54.00, 69.00] | 61.00 [53.00, 68.00] | 66.00 [58.00, 71.00] | <0.001 |
| Smoking, n (%) | 198 (22.27) | 126 (19.35) | 72 (30.25) | 0.001 |
| Frequent urination, n(%) | 204 (22.95) | 159 (24.42) | 45 (18.91) | 0.101 |
| Chest distress, n(%) | 242 (27.22) | 91 (13.98) | 151 (63.45) | <0.001 |
| Chest pain, n(%) | 114 (12.82) | 44 ( 6.76) | 70 (29.41) | <0.001 |
| Palpitation, n (%) | 50 ( 5.62) | 28 ( 4.30) | 22 ( 9.24) | 0.008 |
| Fatigue, n(%) | 33 ( 3.71) | 29 ( 4.45) | 4 ( 1.68) | 0.082 |
| Vomiting, n (%) | 36 ( 4.05) | 35 ( 5.38) | 1 ( 0.42) | 0.002 |
| Chills, n (%) | 11 ( 1.24) | 10 ( 1.54) | 1 ( 0.42) | 0.322 |
| Nausea, n (%) | 47 ( 5.29) | 20 ( 3.07) | 27 (11.34) | <0.001 |
| Fever, n (%) | 86 ( 9.67) | 67 (10.29) | 19 ( 7.98) | 0.367 |
| Hypertension, n (%) | 482 (54.22) | 323 (49.62) | 159 (66.81) | <0.001 |
| WBC (10^9^/L) | 7.08 [5.75, 8.54] | 7.08 [5.65, 8.57] | 7.22 [6.07, 8.41] | 0.304 |
| Neutrophil (10^9^/L) | 4.00 [3.00, 5.18] | 4.00 [3.00, 5.07] | 4.26 [3.35, 5.23] | 0.001 |
| Lymphocytes (10^9^/L) | 2.00 [1.48, 2.33] | 2.00 [1.43, 2.27] | 1.97 [1.56, 2.46] | 0.072 |
| Monocyte (10^9^/L) | 0.59 [0.45, 0.80] | 0.60 [0.45, 0.85] | 0.59 [0.46, 0.73] | 0.274 |
| Eosinophil (10^9^/L) | 0.20 [0.11, 0.30] | 0.20 [0.10, 0.30] | 0.20 [0.11, 0.30] | 0.225 |
| Basophil (10^9^/L) | 0.03 [0.02, 0.04] | 0.03 [0.02, 0.04] | 0.03 [0.02, 0.05] | 0.172 |
| RBC (10^12^/L) | 4.51 [4.00, 5.00] | 4.36 [4.00, 5.00] | 4.68 [4.24, 5.01] | <0.001 |
| Hb (g/L) | 134.00 [121.00, 145.00] | 132.00 [120.50, 144.00] | 136.50 [125.00, 147.00] | 0.002 |
| PLT (10^9^/L) | 241.00 [200.00, 284.00] | 244.00 [200.00, 287.00] | 235.00 [197.75, 268.00] | 0.098 |
| RDW (%) | 41.00 [38.80, 43.10] | 40.50 [38.40, 43.00] | 41.85 [39.60, 44.00] | <0.001 |
| TG (mmol/L) | 1.62 [1.00, 2.30] | 1.45 [1.00, 2.12] | 2.00 [1.36, 2.73] | <0.001 |
| TC (mmol/L) | 4.00 [3.45, 4.89] | 3.90 [3.42, 4.38] | 4.64 [3.97, 5.39] | <0.001 |
| LDL-C (mmol/L) | 2.86 [2.13, 3.37] | 3.00 [2.10, 3.41] | 2.64 [2.18, 3.25] | 0.077 |
| HDL-C (mmol/L) | 1.00 [0.90, 1.17] | 1.00 [0.91, 1.16] | 1.00 [0.88, 1.18] | 0.294 |
| ApoA1 (g/L) | 1.08 [1.00, 1.28] | 1.03 [1.00, 1.26] | 1.17 [1.05, 1.31] | <0.001 |
| ApoB (g/L) | 1.00 [0.82, 1.29] | 1.00 [0.76, 1.20] | 1.20 [0.98, 1.42] | <0.001 |
| TP (g/L) | 66.80 (5.37) | 66.53 (5.41) | 67.54 (5.22) | 0.013 |
| ALB (g/L) | 39.10 [36.92, 41.60] | 39.00 [37.00, 41.42] | 39.40 [36.76, 41.89] | 0.379 |
| AST (U/L) | 20.10 [16.40, 25.00] | 20.00 [16.00, 24.55] | 21.00 [17.80, 26.37] | 0.026 |
| ALT (U/L) | 19.00 [14.20, 27.40] | 19.00 [14.20, 26.35] | 20.05 [14.22, 30.87] | 0.163 |
| LDH (U/L) | 177.00 [156.90, 205.00] | 177.99 [155.00, 204.18] | 176.00 [159.17, 207.00] | 0.581 |
| GGT (U/L) | 30.40 [19.60, 44.87] | 29.32 [18.90, 44.50] | 31.65 [22.05, 45.13] | 0.043 |
| Cr (umol/L) | 75.20 [62.70, 92.00] | 71.00 [60.00, 86.00] | 86.95 [73.20, 108.45] | <0.001 |
| Urea (mmol/L) | 5.30 [4.10, 6.90] | 5.03 [4.00, 6.59] | 5.74 [4.61, 7.41] | <0.001 |
| UA (umol/L) | 357.10 [294.00, 427.00] | 347.90 [285.00, 417.95] | 385.28 [320.29, 463.96] | <0.001 |
| cTn (pg/mL) | 0.01 [0.01, 5.03] | 0.01 [0.01, 0.04] | 0.08 [0.01, 11.90] | <0.001 |
| Myo(ng/mL) | 29.40 [21.10, 42.50] | 28.00 [21.00, 40.98] | 34.39 [25.14, 48.83] | <0.001 |
| CK-MB (ng/mL) | 2.51 [1.25, 11.80] | 2.00 [1.04, 11.00] | 7.84 [1.72, 13.09] | <0.001 |
| TSH (μIU/mL) | 1.62 [1.00, 2.35] | 1.52 [1.00, 2.30] | 1.77 [1.09, 2.59] | 0.001 |
| FT4 (pmol/L) | 11.86 [10.00, 13.47] | 12.00 [10.00, 14.00] | 11.00 [10.00, 12.57] | <0.001 |
| FT3 (pmol/L) | 4.54 [4.00, 5.00] | 4.45 [4.00, 5.00] | 4.64 [4.25, 5.11] | 0.001 |
| APTT (s) | 34.50 [32.20, 37.00] | 34.60 [32.10, 37.00] | 34.30 [32.32, 36.70] | 0.726 |
| PT (s) | 12.80 [12.10, 13.10] | 12.80 [12.00, 13.00] | 12.80 [12.30, 13.30] | 0.018 |
| TT (s) | 17.40 [16.80, 18.10] | 17.20 [16.60, 18.00] | 17.60 [17.10, 18.20] | <0.001 |
| Fbg (g/L) | 3.46 [3.00, 4.10] | 3.37 [3.00, 4.00] | 3.78 [3.20, 4.24] | <0.001 |
| DD (mg/L) | 0.27 [0.13, 0.55] | 0.24 [0.01, 0.52] | 0.34 [0.23, 0.60] | <0.001 |
| CRP (mg/L) | 0.83 [0.30, 5.00] | 0.40 [0.30, 3.63] | 4.03 [1.69, 5.00] | <0.001 |
| HbA1c (%) | 7.95 [6.80, 9.72] | 8.07 [6.90, 10.00] | 7.57 [6.62, 8.81] | <0.001 |
| HCY (μmol/L) | 10.32 [8.84, 12.56] | 10.00 [8.48, 12.07] | 11.23 [9.74, 13.46] | <0.001 |

**Abbreviations:** CHD, coronary heart disease; IQR, interquartile range; SD, standard deviation; WBC, white blood cell count; RBC, red blood cell count; Hb, hemoglobin; PLT, platelet count; RDW, red cell distribution width; TG, triglycerides; TC, total cholesterol; LDL-C, low-density lipoprotein cholesterol; HDL-C, high-density lipoprotein cholesterol; ApoA1, apolipoprotein A1; ApoB, apolipoprotein B; TP, total protein; ALB, albumin; ALT, alanine aminotransferase; AST, aspartate aminotransferase; LDH, lactate dehydrogenase; GGT, gamma-glutamyl transferase; Cr, creatinine; UA, uric acid; cTn, cardiac troponin; Myo, myoglobin; CK-MB, creatine kinase-MB; TSH, thyroid-stimulating hormone; FT3, free triiodothyronine; FT4, free thyroxine; APTT, activated partial thromboplastin time; PT, prothrombin time; TT, thrombin time; Fbg, fibrinogen; DD, D-dimer; CRP, C-reactive protein; HbA1c, glycated hemoglobin; HCY, homocysteine.
